# Prophylactic Use of Continuous Positive Airway Pressure to Operative Lung During One-Lung Ventilation Can Minimize Bleomycin Pulmonary Toxicity: A Report of Two Cases

**DOI:** 10.7759/cureus.36150

**Published:** 2023-03-14

**Authors:** Nicholas Cavanaugh, Sudhakar Subramani, Tyler J Foster, Satoshi Hanada

**Affiliations:** 1 Department of Anesthesia, University of Iowa Hospitals and Clinics, Iowa City, USA; 2 Department of Anesthesia, MercyOne Des Moines Medical Center, Des Moines, USA

**Keywords:** video-assisted thoracoscopic surgery, pulmonary toxicity, thoracic surgery, thoracic anesthesia, bleomycin, one-lung ventilation

## Abstract

Bleomycin, a common antineoplastic agent, is known to cause bleomycin pulmonary toxicity when the lungs are exposed to a high fraction of inspired oxygen (FiO_2_) level. Thus, intraoperative one-lung ventilation (OLV) is challenging in a patient with bleomycin treatment because maintaining high FiO_2_ during OLV is a common practice in thoracic surgery to ensure adequate oxygenation while providing adequate lung isolation. We report two thoracic surgical cases where prophylactic continuous positive airway pressure (CPAP) was applied on the non-dependent lung during OLV while limiting FiO_2_ to prevent postoperative respiratory complications.

## Introduction

A major concern for the anesthesiologist during one-lung ventilation (OLV) is maintaining adequate oxygenation while providing adequate lung isolation. There are many strategies to limit hypoxia during OLV, including using positive end-expiratory pressure (PEEP) on the dependent lung, continuous positive airway pressure (CPAP) on the non-dependent lung, and high fraction of inspired oxygen (FiO_2_) concentrations [1]. In a patient who has been treated with bleomycin, intraoperative OLV is challenging because, in this patient population, it is crucial during surgery to limit FiO_2_ to prevent postoperative respiratory complications [2,3]. Bleomycin, a common antineoplastic agent, is known to cause pulmonary toxicity when the lungs are exposed to high FiO_2_ concentrations [4]. In this two-case series, we discuss the anesthetic considerations when using OLV in patients previously treated with bleomycin and propose an effective way to minimize excessive oxygen exposure by applying CPAP with low FiO_2_ to the non-dependent lung.

## Case presentation

Case 1

A 28-year-old male with a history of Hodgkin’s lymphoma was scheduled for a video-assisted thoracoscopic surgery (VATS) for a left upper lobe biopsy. Of concern, he had recently finished his chemotherapy regimen, which included bleomycin, just 11 days before his scheduled surgery. Preoperative pulmonary testing included pulmonary function testing, which revealed a diffusing capacity of the lungs for carbon monoxide (DLCO) of 60% predicted.

**Figure 1:**
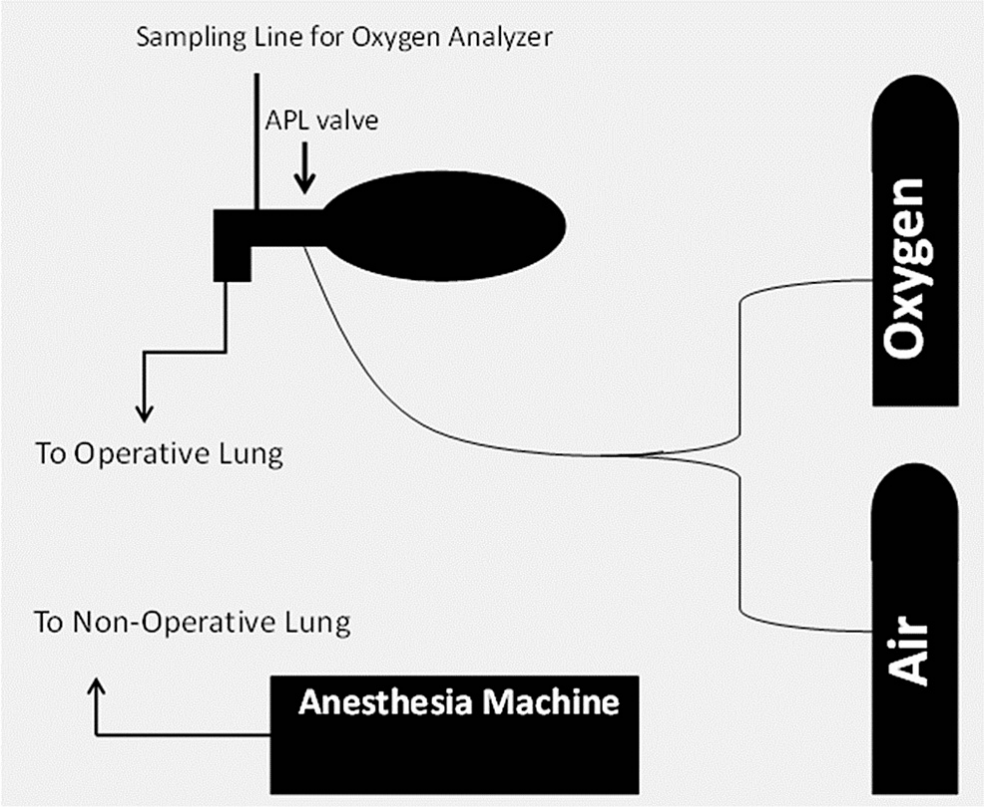
Prophylactic use of CPAP to the operative lung A separate circuit was used to apply CPAP, which included an air source, an oxygen source, and a gas sampling analyzer to adjust the fraction of inspired oxygen. CPAP, continuous positive airway pressure; APL, adjustable pressure limiting

Intraoperatively, the patient was pre-oxygenated with 25% FiO_2_ with 5 cmH_2_O PEEP prior to anesthesia induction and a double-lumen endotracheal tube was placed. A fiberoptic scope was used to confirm the position of the double-lumen tube (DLT). After initiating OLV, a PEEP of 5 cmH_2_O with FiO_2_ of 21-25% was introduced to the dependent non-operative lung, and a CPAP of 3 cmH_2_O with FiO_2_ of 21-25% was applied to the non-dependent lung. The CPAP was applied via a separate circuit, which included an air source, an oxygen source, a separate gas sampling analyzer, and an adjustable pressure valve (Figure 1); hence, the FiO_2_ of the CPAP flow was measurable and adjustable. Saturation of peripheral oxygen (SpO_2_) remained above 90% during the procedure, and partial pressure of oxygen (PaO_2_) was 56 mmHg after 30 minutes of OLV. At the end of the surgery, the patient was successfully extubated in the operation room to room air without hypoxia. FiO_2_ to either lung was kept at or below 25% throughout the case. An arterial blood gas drawn six hours postoperatively revealed PaO_2_ of 90 mmHg. The patient had an uneventful postoperative course and was discharged on postoperative Day 1.

Case 2

A 35-year-old male with a history of metastatic Hodgkin’s lymphoma was scheduled to undergo a VATS for a left lower lobe biopsy. He had previously received six cycles of bleomycin, with the last dose being 23 months prior, along with multiple radiation treatments to his mediastinum. Preoperative chest computed tomography (CT) revealed stable scattered ground-glass airspace disease and pulmonary function testing revealed a DLCO of 74% predicted. He denied having any respiratory symptoms of dyspnea or cough in the preoperative area.

Prior to anesthesia induction, we implemented 5 cmH_2_O PEEP of room air for two minutes to maximize functional residual capacity. During intubation, a nadir SpO_2_ of 86% was observed, which was rapidly corrected with manual ventilation using a FiO_2_ of 0.21-0.25. A fiberoptic scope was used to confirm the position of the DLT. Arterial blood gases were drawn at critical intraoperative times as outlined in Table 1. After initiating OLV, a PEEP of 5 cmH_2_O with FiO_2_ of 21-25% was introduced to the dependent non-operative lung. The same setup described in the first case was used to add 5 cmH_2_O of CPAP to the non-dependent operative lung. A separate gas flow sampling setup confirmed that FiO_2_ was kept at or below 25%. After 30 minutes into OLV, a repeat arterial blood gas showed a PaO_2_ of 67 mmHg. Throughout the procedure, FiO_2_ to either lung was kept at or below 25% while continuous pulse oximetry was maintained between 91% and 98%. At the end of the surgery, the patient was extubated in the operating room to room air without hypoxia. The patient did not develop any respiratory sequelae and was discharged home on postoperative Day 3.

**Table 1:** Arterial blood gas values at critical times of cases PaO2, partial pressure of oxygen in arterial blood; PaCO2, partial pressure of carbon dioxide in arterial blood

	Case 1		Case 2	
Time Collected	PaO_2_	PaCO_2_	PaO_2_	PaCO_2_
Intraoperative: Two-lung ventilation after anesthesia induction (mmHg)	105	37	160	35
One-Lung Ventilation at 30 minutes (mmHg)	67	45	56	43
Postoperative: 6 Hours after extubation (mmHg)	80	40	90	36

## Discussion

In the two case reports presented, our intended outcome was to limit the risk of developing bleomycin pulmonary toxicity by minimizing intraoperative exposure to oxygen. CPAP with low FiO_2_ to the dependent lung was applied to keep the lowest FiO_2_ possible to maintain adequate oxygenation during OLV; thus the risk of postoperative bleomycin pulmonary toxicity could be minimized.

To understand why hyperoxia should be avoided in patients treated with bleomycin, it is pertinent to understand the basic mechanism of how bleomycin damages the lung. Bleomycin pulmonary toxicity is a direct consequence of free radical damage caused by bleomycin, iron, and oxygen forming a complex that cleaves cellular deoxyribonucleic acid (DNA). The lungs are at a higher risk of bleomycin-free radical damage because they lack the enzyme bleomycin hydrolase, which inactivates bleomycin. Reactive oxygen radicals can cause direct cellular toxicity, which could precipitate the release of inflammatory mediators such as prostaglandins, leukotrienes, and other cytokines. Direct damage to alveolar epithelial cells can lead to myofibroblast proliferation and increased collagen synthesis, resulting in pulmonary fibrosis [5].

It is imperative that the anesthesiologist identify patients at high risk for bleomycin pulmonary toxicity. Two major risk factors, which are linked to hyperoxia exposure include (1) pre-existing pulmonary toxicity due to bleomycin and (2) prior exposure to bleomycin within the past two months [6]. Recent recommendations state that patients with at least one major risk factor should be maintained on the least amount of FiO_2_ to maintain SpO_2_ greater than 90%. Clinical symptoms of a pre-existing pulmonary disease after bleomycin exposure include dyspnea, tachypnea, or a non-productive cough. Preoperative testing is an important step to recognize these patients who may have preexisting pulmonary damage. Chest CT findings may include diffuse alveolar damage, such as diffuse airspace consolidation and ground-glass opacifications, and pulmonary function tests may manifest as a decreased DLCO.

There are no current randomized studies that have definitively determined safe FiO_2_ concentrations during OLV in patients who have been previously treated with bleomycin. However, many researchers have correlated high FiO_2_ concentrations with lung toxicity. The concept of minimizing FiO_2_ concentration during the perioperative setting was first explored by Goldiner et al. in 1978 [2]. Further study has recommended that the anesthesiologist should use the lowest FiO_2_ to maintain adequate oxygenation [7]. Several retrospective studies have examined postoperative respiratory complications in these patients and compared these to intraoperative FiO_2_ levels [8,9]. Of special concern, Ingrassia, et al. published a case report where a bleomycin-exposed patient developed postoperative acute respiratory distress after a brief high FiO_2_ (up to 71%) exposure during intraoperative OLV [5]. All of these studies have reached the same conclusion: that a FiO_2_ of less than 30% minimizes the risk of developing bleomycin pulmonary toxicity.

We present two cases where OLV was successfully implemented with limited FiO_2_ by applying CPAP with low FiO_2_ to the non-dependent lung. Early initiation of CPAP during OLV might attenuate a ventilation-perfusion (V/Q) mismatch by minimizing local hypoxia. The use of CPAP during OLV has proven successful in other case reports; however, there is little data on what amount of CPAP or FiO­_2_ can be used successfully [10]. In our case series, the application of 3 to 5 cmH_2_O of CPAP with FiO_2_ of 21% to 25% prevented systemic hypoxia during OLV. The unique feature of our setup is the creation of a separate CPAP circuit where FiO_2_ is measurable and adjustable (Figure 1). This setup is inexpensive and can be easily reproduced, even in centers with limited resources. The disadvantage of the application of CPAP is limiting surgical field exposure during a VATS; however, this can be minimized by using low amounts of CPAP such as the 3-5 cm H_2_O used in our two cases. Hypoxemia may still develop despite PEEP and CPAP, especially in the setting of minimal supplemental oxygen. Hence, it is critical that close monitoring with continuous pulse oximetry and frequent arterial blood gases should guide therapy to prevent hypoxemia.

## Conclusions

In conclusion, we are in agreement that the anesthesiologist should use the lowest FiO_2_ to maintain adequate oxygenation for preventing bleomycin pulmonary toxicity. Since there are currently no studies that determine what percentage of FiO_2_ is safe to prevent bleomycin pulmonary toxicity, alternative strategies like PEEP and CPAP should be considered to minimize inspired oxygen requirements during OLV. The current case series implies that the application of CPAP with limiting inspired oxygen to the non-dependent lung is useful for preventing hypoxemia.
